# Inducing Tolerance to Abiotic Stress in *Hordeum vulgare* L. by Halotolerant Endophytic Fungi Associated With Salt Lake Plants

**DOI:** 10.3389/fmicb.2022.906365

**Published:** 2022-05-20

**Authors:** Mahdieh S. Hosseyni Moghaddam, Naser Safaie, Saleh Rahimlou, Niloufar Hagh-Doust

**Affiliations:** ^1^Department of Plant Pathology, Faculty of Agriculture, Tarbiat Modares University, Tehran, Iran; ^2^Institute of Ecology and Earth Sciences, University of Tartu, Tartu, Estonia; ^3^Center of Mycology and Microbiology, University of Tartu, Tartu, Estonia

**Keywords:** crop production, environmental stress, halotolerant fungal endophytes, growth promotion, reactive oxygen species, stress tolerance

## Abstract

A characteristic trait of plants living in harsh environments is their association with fungal endophytes, which enable them to survive under extreme stress. Abiotic stress resistance in agro-ecosystems, particularly in arid and semi-arid regions, can be increased by inoculating these fungal endophytes on plants other than their original hosts. The present study is therefore focused on the possible role of three halotolerant endophytic fungi, i.e., *Periconia macrospinosa*, *Neocamarosporium goegapense*, and *N. chichastianum*, isolated from roots of salt lake plants growing in the central desert of Iran, in alleviating the adverse effects of salinity and drought stresses on barley under greenhouse conditions. To perform this experiment, a randomized block design was applied with three factors: fungi (four levels including three halotolerant endophytic species and control), salinity (three levels including 8, 12, and 16 dS/m), and drought (four levels including 100, 80, 60, 40 percent field capacity). All plants were measured for growth characteristics, chlorophyll concentration, proline content, and antioxidant enzyme activities. A three-way analysis of variance indicated that all three fungal endophytes, to varying extents, induced the barley plants’ resistance to salinity and drought, and their combined effects. Additionally, we found that fungal endophytes were more effective when the barley plants were subjected to higher levels of salinity and drought. Under the stress of salinity and drought, a strong relationship between inoculation of fungal endophytes and enhancement of biomass, shoot length, chlorophyll concentration, proline content, and activity of catalase, peroxidase, and superoxide dismutase was indicated. We discussed that increased root growth, proline content, and antioxidant enzyme activity are the main physiological and biochemical mechanisms causing stress resistance in barley plants inoculated with endophytes. Our research findings illustrate that fungal endophytes have a substantial potential for increasing abiotic stress tolerance in barley plants, which can be applied in agricultural ecosystems.

## Introduction

Environmental challenges such as drought and salinity impose the greatest threat to plants growing on irrigated land which generates about 40% of the world’s food (Chaves et al., 2003; Pimentel et al., 2004; Omar et al., 2009; Alikhani et al., 2013). Morphological changes, restriction of photosynthesis, growth reduction, disruption in the activities of the various enzymes, influence on the formation of reactive oxygen species (ROS), and performance imperfection are significant adverse effects of such environmental stress on crops (Yildirim et al., 2015; Sahin et al., 2018). Scientists around the world are searching for innovative methods to maintain crop yields in stressful conditions. Numerous studies have shown that plant-associated microorganisms increase plant resistance to environmental stress (Soussi et al., 2016; Bonatelli et al., 2021). The research to date has mainly focused on fungal and bacterial symbionts, such as mycorrhiza and nitrogen-fixing bacteria, and little is known about the potential benefits of other free-living endophytic microorganisms. However, some researchers have reported endophytes as one of the key plant-associated microorganisms involved in helping their hosts to increase growth and overcome biotic and abiotic stress (Khan et al., 2011; Piernik et al., 2017; Fouda et al., 2019; El-Sersawy et al., 2021).

Recent studies have indicated that endophytic microorganisms associated with plant species growing in harsh environments could induce significant abiotic stress tolerance in plants other than their original hosts (Redman et al., 2011; Moghaddam et al., 2021). These microorganisms increase environmental stress tolerance in their host plants by several mechanisms, such as raising water-use efficiency, enhancing antioxidant enzyme activity, the adjustment in ion transport and metabolic changes, modulation of phytohormones, etc. (Baltruschat et al., 2008; Lata et al., 2018). Bacterial endophytes associated with halophytic plants, i.e., *Arthrocnemum macrostachyum* and *Spergularia marina*, ameliorate salinity tolerance of *Vicia faba* by improvement of enzymatic and non-enzymatic antioxidant accumulation (Mahgoub et al., 2021). Endophytic ascomycete *Curvularia* sp. isolated from halophytic *Suaeda salsa* conveys salinity stress tolerance to poplar trees *Populus tomentosa* (Pan et al., 2018). Poplar trees inoculated with *Curvularia* sp. indicated higher levels of antioxidant enzymes and proline than non-inoculated plants. *Thermomyces* fungal endophyte isolated from extreme hot desert-adapted plants eliminates the adverse effects of heat stress on cucumber plants by increasing root length, improving photosystem II efficiency, photosynthesis rate, and water use efficiency relative to endophyte-free plants (Ali et al., 2018). In light of these studies isolating endophytic microorganisms associated with desert plants and studying their role in inducing tolerance to abiotic stress are of great biotechnological importance, especially in barren lands and saline environments.

Barley (*Hordeum vulgare*), as a monocotyledonous plant species, is the fourth most commonly produced cereal after maize, rice, and wheat. Therefore, studies on the methods of how to increase barley plants’ resistance to abiotic stress are crucial for agricultural research. During the last decade, several studies have investigated the effect of fungal endophytes on barley plants’ increasing resistance to salinity and drought stresses (Alikhani et al., 2013; Bagheri et al., 2013; Chen et al., 2018). Inoculation of *Epichloë*, an asexual endophytic fungus, significantly alleviated adverse effects of salinity stress on barley plants by promoting nutrient absorption and adjusting the ionic balance (Song et al., 2015). Improvement of ascorbic acid levels and antioxidant enzyme activity were observed in barley plants inoculated with *Piriformospora indica* under salinity stress (Baltruschat et al., 2008). Ghabooli et al. (2013) reported that *P. indica*, as a mutualistic root endophytic fungus, increases growth and salinity tolerance in barley plants by enhancing the K^+/^Na^+^ and Ca^2+^/Na^+^ ratios, sugars, and free amino acids. Endophytic fungus *Epichloë bromicola* significantly increased salinity stress in *H. brevisubulatum* by increasing the conversion of putrescine to spermidine and spermine as well as improving shift ability of free and soluble conjugated forms of polyamines to insoluble forms (Prema Sundara Valli and Muthukumar, 2018).

We previously indicated that three halotolerant endophytic fungal species, i.e., *Periconia macrospinosa*, *Neocamarosporium goegapense*, and *N. chichastianum*, isolated from the roots of Salt Lake plants growing in the central desert of Iran increase dicot plants’ resistance to salinity and drought stresses (Moghaddam et al., 2021). This study aimed to investigate the potential benefits of these endophytic fungi in inducing the same abiotic stress tolerance in monocots. Fungal treatments including the three mentioned species and control plants (lacking endophytes) – under different salinity, drought, and their combination stress levels were applied to barley plants in a greenhouse experiment. For understanding the barley plant’s reaction to different levels of salinity and drought stress, several important physiological and biochemical markers such as proline content of leaf and antioxidant enzymatic activities in the presence/absence of endophytic fungi were measured. Furthermore, growth parameters and leaf chlorophyll concentration were measured.

## Materials and Methods

### Greenhouse Experimental Design

*Hordeum vulgare* L. (barley) was used as a model plant for salinity and drought stress tolerance assays. Salt-sensitive barley seeds (Reyhan 03, Iran) were surface-sterilized for 10 min in 0.25 percent sodium hypochlorite, followed by rinsing with water (Baltruschat et al., 2008). The surface-sterilized seeds were germinated at 24°C on sheets of Whatman filter paper, which were soaked in distilled water in sterile Petri dishes. After two days, the germinated seeds were transferred to pots containing 200 g of 2:1 autoclaved mixture of soil and peat (v/v) (pH = 6.7; EC = 1.1 dS/m). This experiment was conducted using a random block design, with 144 pots divided up into 36 pots for each fungal species treatment, and 36 pots for control treatments.

Three halotolerant fungal endophyte species, i.e., *Periconia macrospinosa*, *Neocamarosporium goegapense*, and *N. chichastianum*, isolated in our previous study (Moghaddam et al., 2021) were propagated in Potato Dextrose Broth (PDB; Merck, Germany) for about ten days at 28°C, shaking at 180 rpm, in dark conditions. The pots were inoculated with fungal endophytes by mixing 200 g of the potting substrate with 2 g of fresh mycelium. The pots were maintained in a greenhouse at 27:19°C day: night cycle, 45–55% relative humidity, and a photoperiod of 16 h.

Barley plants were exposed to salinity and drought 10 days after inoculation. Salt lake water (31.4% Na^+^; 0.3% K; 0.9% Mg; 0.3% Ca, 12.7% Cl; 0.8% SO_4_; and 1.29% CO_3_) from Hoz-e Soltan (35.00083333, 50.99250000) was used to induce salinity stress on endophyte-colonized plants as well as on the endophyte-free control plants. Salinity stress was applied at three levels of Electrical conductivity to the barley pots, including S_1_ = 8 dS/m (without yield reduction), S_2_ = 12 dS/m (25 percent yield reduction), and S_3_ = 16 dS/m (50 percent yield reduction). In order to attain different salinity levels, the salt lake water was diluted with distilled water. Barley plants were exposed to drought stress at four different levels, including D_1_ = 100%, D_2_ = 80%, D_3_ = 60%, and D_4_ = 40% of field capacity (FC). In order to determine the field capacity, soil samples of 200 g were taken at the time of filling plastic pots, stored in plastic bags, and transported to the laboratory. A 24-hour incubation at 105°C was then performed on these soil samples. By weighing and averaging these oven-dried samples, we determined the soil moisture content at the time of sowing the barley seeds. In order to estimate the saturation percentage of the oven-dried soil samples, distilled water was used to make a paste that was completely saturated.

The following equation was used to determine the field capacity:


Fieldcapacity=Saturationpercentage/2

A drought treatment was calculated as 100, 80, 60, and 40 percent field capacities. By using a balance, pots were weighed to determine the soil’s field capacity. Plants were only watered if the capacity of the field decreased (Saeed et al., 2016). All experiments were applied in triplicates.

### Confirmation of the Colonization of Barley Roots by Fungal Endophyte

We examined the roots and confirmed the internal plant colonization by the inoculated endophytic fungi as follows. Root samples were washed under tap water for 10 minutes and cut into 0.5-1 cm pieces. Root fragments were subjected to a three-step surface sterilization process including dipping in ethanol/water (70:30) for 2 min, followed by sodium hypochlorite/water (4:96) for 90 s, ethanol/water (70:30) for 2 min, and a final rinse in sterile distilled water for three times (1 min each). The inner layers of disinfected roots were cultured on PDA (Merck, Darmstadt, Germany), and incubated at 28°C (Hosseyni-Moghaddam and Soltani, 2014). A small amount of water from the final step was also spread on the same media. The control plants were treated in the same way. The isolated fungal species were identified either morphologically (Supplementary Figure 1) or by sequencing the ITS-rDNA region.

### Measurements of Growth Parameters and Chlorophyll Concentration

Six weeks after planting the seeds, the root wet and dry weight and shoot length of the plants were measured. Using a SPAD-502 Chlorophyll meter, we measured the chlorophyll content of all leaf samples (Coste et al., 2010).

### Antioxidant Enzymatic Activity and Proline Content of Leaf Measurements

Two weeks after the pots were stressed by salinity and drought, leaf samples were collected. Liquid nitrogen was used to crush 0.2 g of each leaf sample for homogenization. Then, 3 mL of HEPES-KOH buffer (pH 7.8) containing 0.1 mM EDTA was added to the leaf powder. Supernatants (enzyme extracts) were used for chemical analysis once the final solutions had been centrifuged at 4°C at 16,000 rpm for 15 min (Sahebjamei et al., 2007). A photochemical method was applied to measure superoxide dismutase (SOD) activity in the supernatant (Giannopolitis and Ries, 1977). First, a reaction mixture (3 ml) containing 300 μL enzyme extract, 75 μM nitroblue tetrazollium chloride (NBT), 1 μM riboflavin, 0.1 mM EDTA, 50 mM MHEPES-KOH buffer (pH 7.8), 12 mM L-methionine, and 50 mM Na_2_CO_3_ (pH 10.2), was prepared. Then, using a spectrophotometer (Cintra 6, GBC, Dandenong, Australia), one unit of superoxide dismutase activity was determined as the amount of enzyme needed to inhibit the 50% rate of NBT reduction measured at 560 nm. In a reaction mixture consisting of 500 μl of 10 mM H_2_O_2_, 1400 μl of 25 mM sodium phosphate buffer, and 100 μl of crude enzyme extract, catalase activity was measured. Following the decomposition of H_2_O_2_, absorbance at 240 nm declined. Catalase activity of the extract was expressed as CAT unit: min^–1^ mg^–1^ protein (Cakmak and Horst, 1991). A spectrophotometric measurement of the peroxidase activity was done by following the oxidation of guaiacol (2-methoxyphenol) to tetraguaiacol at 470 nm (Plewa et al., 1991). The reaction mixture (3 mL) contained 500 μl 28 mM guaiacol, 1900 μl 60 mM potassium phosphate buffer (pH 6.1), 500 μl 5 mm H2O2, and 100 μl crude extract. The enzyme activity was expressed as POX unit: min^–1^ mg^–1^ protein.

For the estimation of proline content, each leaf sample (0.2 g) was homogenized in 3 ml sulphosalicylic acid (3% w/v), and then centrifuged at 10,000 rpm for 15 min. A reaction mixture consisted of 2 ml supernatant, 2 ml freshly prepared acid ninhydrin solution, and 2 ml glacial acetic acid which was boiled at 100°C for 1 h. After completion of the reaction in an ice bath, the reaction mixture was extracted with 4 ml of toluene; absorbance was read at 520 nm (Bates et al., 1973).

### Statistical Analyses

For each measured biological parameter of the barley plants, a three-way ANOVA was used to determine the effects of three factors, i.e., fungus, salinity, and drought. LSD tests were used to assess the significance of group differences at a *p* ≤ 0.05 level. Analyses were conducted using statistical packages in R version 3.6.2 (R Core Team, 2021).

## Results

### Colonization of Barley Roots by Halotolerant Endophytic Fungi

By using the same isolation technique, the isolated halotolerant fungal endophytes, i.e., *P. macrospinosa, N. goegapense*, and *N. chichastianum*, were re-isolated from the inoculated barley roots. However, these fungal species were not isolated from the control plants. The confirmation of colonization of barley roots by halotolerant endophytic fungi validated our results from this experiment.

### Growth Promotion of Barley Plants Under Salinity and Drought Stress

A three-way analysis of variance revealed that halotolerant endophytic fungi significantly increased the tolerance of barley plants to salinity, drought, and their combined effects (Tables 1, 2). Compared to the endophyte-free control plants, the barley plants’ growth parameters were all improved (*p* ≤ 0.01; Table 1). Six weeks after planting the seeds, inoculated barley plants indicated 27.6, 30.5, and 24.7% more biomass, root wet weight, and shoot length than their control counterparts, respectively. The results also indicated that the increasing drought or salinity levels negatively affect barley plants’ growth characteristics (*p* ≤ 0.01); however, the interaction effect of these abiotic stress was not significant (Table 1).

**TABLE 1:** Three-way analysis of variance (ANOVA) of the effects of the halotolerant fungal endophytes, *P. macropinosa*, *N. goegapense*, and *N. chichastianum* on the growth parameters and leaf chlorophyll concentration of *Hordeum vulgare* L. (barley) under different levels of salinity and drought stresses.

Variables	DF^a^	Biomass	Root wet weight	Leaf chlorophyll concentration	length shoot
Fungi	3	32.16***	58.03***	2325***	7013***
Drought	3	46.07***	109.83***	5349***	13187***
Salinity	2	12.30***	17.45***	389***	1844***
Fungi:Drought	9	4.60***	8.34***	391***	928***
Fungi:Salinity	6	1.60*	2.66***	45	226***
Drought:Salinity	6	0.02	0.02	0	2
Fungi:Drought:Salinity	18	0.06	0.10	0	6
Residuals	96	4.11	5.89	328	496

Significant codes: 0 ‘***’ 0.01 ‘*’ 0.05 ‘.’ 0.1 ‘’ 1.

*a Degree of freedom.*

*The numbers presented in the table are the sum of squares of the fitted model.*

**TABLE 2:** Three-way analysis of variance (ANOVA) of the effects of the halotolerant fungal endophytes, *P. macropinosa*, *N. goegapense*, and *N. chichastianum* on the activity of antioxidant enzymes including POX, CAT, and SOD, and leaf proline content of *Hordeum vulgare* L. (barley) under different levels of salinity and drought stresses.

Variables	DF^a^	POX	CAT	SOD	Leaf Proline content
Fungi	3	0.09***	0.10***	0.42***	1033***
Drought	3	1.20***	0.59***	2.65***	8044***
Salinity	2	0.10***	0.01***	0.24***	917***
Fungi:Drought	9	0.03***	0.01***	0.11***	464***
Fungi:Salinity	6	0.001***	0.001***	0.03***	49***
Drought:Salinity	6	0.001***	0.0006***	0.003***	4***
Fungi:Drought: Salinity	18	0.0003***	0.001***	0.01***	17***
Residuals	96	0	0.0001	0.0001	3

Significant codes: 0 ‘***’ 0.001 ‘**’ 0.01 ‘*’ 0.05 ‘.’ 0.1 ‘’ 1.

*a Degree of freedom.*

*The numbers presented in the table are the sum of squares of the fitted model.*

**FIGURE 1:**
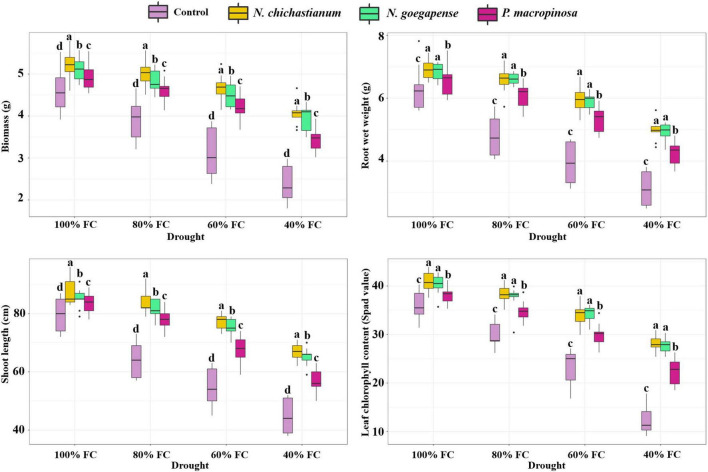
The positive effects of halotolerant endophytic fungi species on drought stress resistance abilities of barley plants. Different levels of drought stress and different fungal treatment are shown on the horizontal axis and colorful boxes, respectively. Various letters on the top of bars display significant differences between mean values of different fungal treatments under the different levels of drought stress at p ≤ 0.05.

Barley plants’ growth parameters were affected by the interaction between halotolerant endophytic fungi and drought stress (*P* ≤ 0.01; Table 1). Under D_1_, D_2_, D_3_, D_4_ drought levels, the biomass of the inoculated barley plants was observed to be increased by 9.2, 23.1, 42, and 61.1 percent compared to the endophyte-free controls (Figure 1). Shoot length also increased by 7.2, 26.5, 34.7, and 41 percent compared to the control plants under D_1_, D_2_, D_3_, D_4_ drought levels (*p* ≤ 0.05; Figure 1). In total, plants inoculated with *N. chichastianum* indicated greater biomass and shoot length than those inoculated with *P. macrospinosa*, and *N. goegapense* across all levels of drought stress (*p* ≤ 0.05; Figure 1). Additionally, endophyte-colonized barley showed 7.4, 34.1, 47.1, and 50.9 percent greater root wet weight compared to endophyte-free controls, under D_1_, D_2_, D_3_, D_4_ drought levels, respectively (Figure 1). Overall, plants inoculated with *Neocamarosporium* species showed greater root wet weight than those inoculated with *P. macrospinosa* (*p* ≤ 0.05; Figure 1).

**FIGURE 2:**
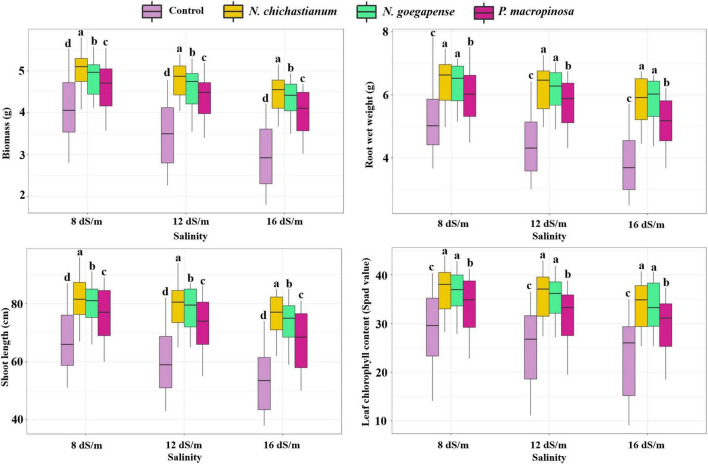
The positive effects of halotolerant endophytic fungi species on salinity stress resistance abilities of barley plants. Different levels of drought stress and different fungal treatment are shown on the horizontal axis and colorful boxes, respectively. Various letters on the top of bars display significant differences between mean values of different fungal treatments under the different levels of salinity stress at p ≤ 0.05.

Growth parameters of barley plants were affected by interactions between halotolerant endophytic fungi and salinity stress (Table 1). Under salinity levels of S_1_, S_2_, and S_3_, the biomass of inoculated barley plants was increased by 17, 31.7, and 42.9 percent in comparison with endophyte-free control plants (Figure 2). The shoot length of the barley plants was observed to be increased by 17, 25.9, and 32.9 percent compared to the endophyte lacking controls under S_1_, S_2_, and S_3_ salinity levels (Figure 2). Overall, *N. chichastianum*-inoculated plants indicated greater biomass and shoot length compared to the plants associated with *P. macrospinosa*, and *N. goegapense*, across all salinity stress levels (*p* ≤ 0.05; Figure 2). Under salinities of S_1_, S_2_, and S_3_, the endophyte-colonized barley plants indicated 18.9, 33.4, and 42.8 percent greater root wet weight than the endophyte-free plants, respectively (*p* ≤ 0.05; Figure 2). The barley plants inoculated with *Neocamarosporium* species indicated greater root wet weight than those inoculated with *P. macrospinosa* at all levels of salinity stress (*p* ≤ 0.05; Figure 2).

The outcomes indicated that a three-way interaction between drought, salinity, and fungus failed to significantly influence the growth characteristics of barley plants (Table 1). However, higher growth parameters were recorded in inoculated barley plants compared to the control plants. Additionally, the halotolerant endophytic fungi were more effective at improving barley plants’ growth parameters when the plants were exposed to high levels of drought or salinity stress (Figures 1, 2).

### Effects of Halotolerant Endophytic Fungi on Leaf Chlorophyll Concentration of Barley Plants Under Salinity and Drought Stress

A three-way ANOVA study demonstrated that halotolerant endophytic fungi species significantly increased leaf chlorophyll concentration compared to the control plants lacking fungal endophytes (*P* ≤ 0.01; Table 1). Six weeks after planting the seeds, plants colonized with halotolerant endophytic fungi indicated greater chlorophyll content in comparison with endophyte-free plants. The results also showed that increasing drought or salinity levels negatively affect barley plants’ chlorophyll concentration (*p* ≤ 0.01); however, the interaction effect of these abiotic stress was not significant (Table 1).

Chlorophyll content of leaves was positively affected by halotolerant endophytic fungi under different levels of drought stress (*p* ≤ 0.01; Table 1). Compared to the endophyte-free barley plants, plants colonized with endophytes showed 10, 23, 42.8, and 110.4 percent higher chlorophyll contents at D_1_, D_2_, D_3_, D_4_ drought levels (*p* ≤ 0.05; Figure 1). Contrary to our expectations, the presence of halotolerant endophytic fungi did not significantly affect chlorophyll concentrations in the leaves under salinity stress (Table 1). Nevertheless, plants inoculated with *Neocamarosporium* species had higher chlorophyll content than plants inoculated with *P. macrospinosa*, irrespective of the degree of drought stress (*p* ≤ 0.05; Figure 1). Furthermore, results indicated that halotolerant fungal endophytes were more effective at improving leaf chlorophyll content when barley plants were subjected to a higher level of drought stress (Figure 1).

The effects of the three-way interactions between drought, salinity, and fungus on leaf chlorophyll concentration of barley plants were not significant (Table 1). However, endophyte-colonized plants had higher chlorophyll levels than endophyte-free plants.

### Effects of Halotolerant Fungal Endophytes on Leaf Proline Content of Barley Plants Under Salinity and Drought Stress

**FIGURE 3:**
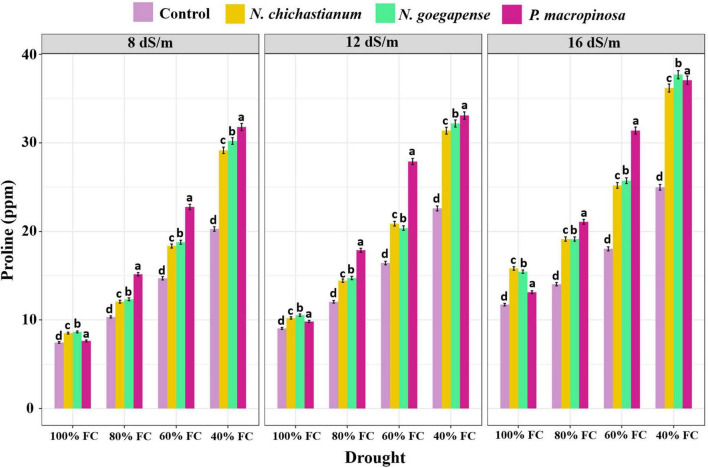
The effects of halotolerant endophytic fungi species on leaf proline content of barley plants, under salinity and drought stress. Different levels of drought stress and different fungal treatment are shown on the horizontal axis and colorful bars, respectively. Various letters on the top of bars display significant differences between mean values of different fungal treatments under the different levels of salinity and drought stress at p ≤ 0.05.

Variance partitioning analysis indicated that halotolerant fungal endophytes significantly affected proline content of barley plants (*p* ≤ 0.01, Table 2). Barley plants associated with *P. macrospinosa, N. goegapense*, and *N. chichastianum* showed 47.8, 35.9, and 32.8 percent higher proline levels than their endophyte-free counterparts (Figure 3). Moreover, increasing salinity increased leaf proline content of barley plants across the experiment. Similar patterns were observed in plants subjected to drought stress (Table 2 and Figure 3).

Increasing salinity or drought stress levels significantly increased leaf proline content of barley plants across the experiment. Moreover, the interaction effects of salinity and drought stress had significant effects on proline content (*p* ≤ 0.01, Table 2). Under S_1_, S_2_, and S_3_ salinity levels, the proline content of inoculated barley plants was observed to be increased by 34.9, 36, and 43.9 percent compared to endophyte lacking controls, respectively. Further, interaction between halotolerant fungal endophytes and drought stress significantly affected the proline content of barley plants (p ≤ 0.01, Table 2). When the endophyte-colonized barley plants were subjected to D_1_, D_2_, D_3_, D_4_ drought levels, their proline content was 17.8, 33.5, 43.2, and 46.7 percent higher than the endophyte-free controls, respectively (*p* ≤ 0.05; Figure 3).

Outcomes indicated that the three-way interactions between salinity, drought, and fungus significantly impacted proline content (*p* ≤ 0.01; Table 2). With increasing salinity and drought levels, the halotolerant fungal species were more effective on leaf proline content. Under the lowest levels of salinity and drought stress, inoculation of *P. macrospinosa, N. goegapense*, and *N. chichastianum* increased the proline content of leaf by 2.3, 15.9, and 14.3 percent, respectively, compared to the control plants (*p* ≤ 0.05; Figure 3). While under the highest levels of drought and salinity, the barley plants colonized by *P. macrospinosa, N. goegapense*, and *N. chichastianum* showed a significant increase of proline content of leaf, by 48.4, 50.9, and 44.8 percent, respectively, compared to the control plants (*p* ≤ 0.05, Figure 3). Overall, plants colonized with *N. goegapense* expressed the highest proline content of leaf under the lowest and highest salinity levels and drought stresses (Figure 3).

### Effects of Halotolerant Fungal Endophytes on Antioxidant Enzymatic Activities of Barley Plants Under Salinity and Drought Stress

In barley plants, fungus, salinity, and drought treatments, and their interactions, significantly affected the activity of antioxidant enzymes including SOD, CAT, and POX (*p* ≤ 0.01, Table 2).

**FIGURE 4:**
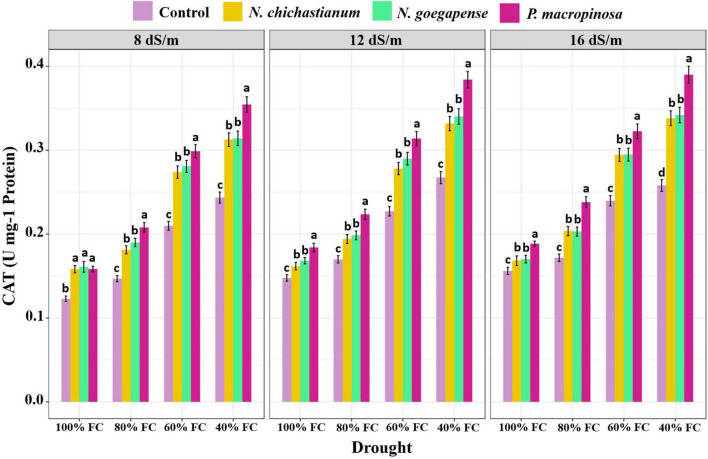
The effects of halotolerant endophytic fungi species on CAT activity of barley plants, under salinity and drought stress. Different levels of drought stress and different fungal treatment are shown on the horizontal axis and colorful bars, respectively. Various letters on the top of bars display significant differences between mean values of different fungal treatments under the different levels of salinity and drought stress at p ≤ 0.05.

**FIGURE 5:**
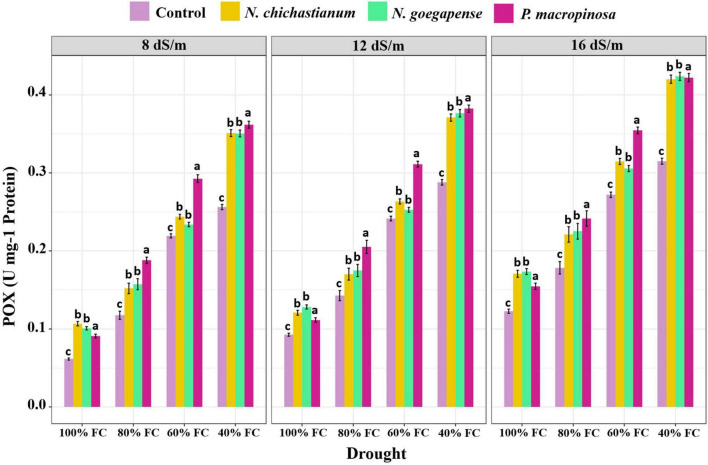
The effects of halotolerant endophytic fungi species on POX activity of barley plants under salinity and drought stress. Different levels of drought stress and different fungal treatment are shown on the horizontal axis and colorful bars, respectively. Various letters on the top of bars display significant differences between mean values of different fungal treatments under the different levels of salinity and drought stress at p ≤ 0.05.

**FIGURE 6:**
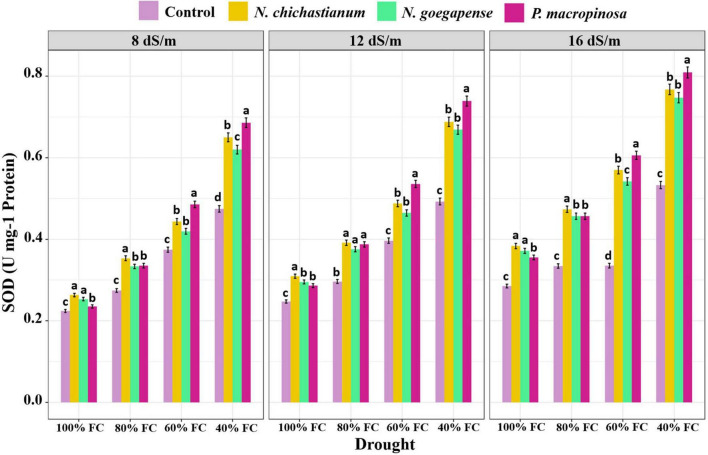
The effects of halotolerant endophytic fungi species on SOD activity of barley plants under salinity and drought stress. Different levels of drought stress and different fungal treatment are shown on the horizontal axis and colorful bars, respectively. Various letters on the top of bars display significant differences between mean values of different fungal treatments under the different levels of salinity and drought stress at *p* ≤ 0.05.

As compared to the endophyte-free controls, plants inoculated with *P. macrospinosa*, *N. goegapense*, and *N. chichastianum* demonstrated 38.2, 25, and 22.6 percent greater CAT activity (Figure 4). The inoculations of P. macrospinosa, N. goegapense, and *N. chichastianum* also enhanced POX activity by 35, 25.8, and 25.9 percent, respectively (Figure 5). In addition, enhancement of SOD activity by 38.6, 29.9, and 35.4 percent, was observed in plants colonized with *P. macrospinosa*, *N. goegapense*, and *N. chichastianum* (Figure 6). The results also showed that increasing salinity or drought stress levels, as well as their interaction effects, significantly increased the SOD, CAT, and POX activities of barley plants (*p* ≤ 0.01, Table 2).

Salinity and fungus interaction significantly affected SOD, CAT, and POX activities (p ≤ 0.01; Table 2). Under S_1_, S_2_, and S_3_ salinity levels, we respectively observed a 25.6, 30.9, and 46.4 percent increase in SOD activity among inoculated barley plants compared to endophyte-free control plants (Figure 6). Barley plants colonized with endophytes also showed 20.6, 25.9, and 33.2 percent greater CAT activity than those not colonized with endophytes, under S_1_, S_2_, and S_3_ salinity levels (*p* ≤ 0.05; Figure 3). Furthermore, under S_1_, S_2_, and S_3_ salinity levels, endophyte-inoculated barley plants respectively indicated 24.9, 28.6, and 33.9 percent higher POX activities compared to the control plants (*p* ≤ 0.05; Figure 5).

Drought and fungus interaction significantly affected SOD, CAT, and POX activities (*p* ≤ 0.01, Table 2). Under D_1_, D_2_, D_3_, D_4_ drought levels, we respectively observed a 21.2, 32.1, 37.1, and 41.6 percent increase in SOD activity among inoculated barley plants compared to the endophyte-free control plants (Figure 6). Barley plants colonized with endophytes also indicated 18.6, 25.5, 30.3, and 34.6 percent greater CAT activity than those not colonized with endophytes, under D_1_, D_2_, D_3_, D_4_ drought levels (*p* ≤ 0.05; Figure 3). Moreover, under D_1_, D_2_, D_3_, D_4_ drought levels, endophyte-inoculated barley plants respectively indicated 39.3, 31.9, 16.9, and 34.2 percent higher POX activities compared to the control plants (*p* ≤ 0.05; Figure 5).

Three-way interactions between drought, salinity, and fungus had significant effects on SOD, CAT, and POX enzymes activity (*p* ≤ 0.01, Table 2). Moreover, fungal endophytes were more influential on barley plants’ antioxidant enzyme activities as salinity and drought increased (Figures 4–6). Under the lowest and highest levels of drought and salinity, barley plants colonized by *P. macrospinosa*, *N. goegapense*, and *N. chichastianum* showed higher SOD, CAT, and POX enzymatic activity than their control counterparts (*p* ≤ 0.05). Under the lowest levels of salinity and drought stresses, *P. macrospinosa, N. goegapense*, and *N. chichastianum* increased the CAT activity by 28.9, 30.9, and 28.8 percent, SOD activity by 1.7, 1.2, and 4.9 percent as well as POX activity by 48.2, 64, and 73.5 percent compared to the controls, respectively (*p* ≤ 0.05; Figures 4–6).

Under the highest levels of salinity and drought, *P. macrospinosa, N. goegapense*, and *N. chichastianum* significantly increased the CAT activity by 51.1, 32, and 30.9 percent, and SOD activity by 51.8, 40.2, 44 percent as well as POX activity by 34, 34.5, and 33.3 percent compared to the control plants, respectively (*p* ≤ 0.05; Figures 4–6).

## Discussion

Drought and salinity threaten agricultural production, especially in arid and saline environments. Increasing food demand requires finding non-chemical ways to enhance plants’ tolerance to salinity and drought. The evidence for endophytes as beneficial microbes has been growing in recent years, confirming their ability to confer certain benefits to their host plants under abiotic stress, such as persistent drought, extreme temperatures, high salinity, and heavy metal toxicity (Singh et al., 2011; Shahabivand et al., 2017; Lata et al., 2018; Alkahtani et al., 2020). In the meantime, the study of endophytes associated with plants living in harsh environments has gained increasing attention, with the hypothesis that these microorganisms are responsible for their host’s tolerance of such conditions (Alkahtani et al., 2020; Mahgoub et al., 2021). Here we indicated that three halotolerant fungal endophytes, i.e., *P. macrospinosa, N. goegapense*, and *N. chichastianum*, isolated from roots of Salt Lake plants growing in the central desert of Iran, increase barley plant’s tolerance to salinity, drought, and their combined effects. Furthermore, we found that these three fungal endophytes are more effective under high levels of drought and salinity stresses. This suggests that endophytic fungi might benefit plants more in adverse conditions. A similar result was observed when dicotyledonous plants inoculated with fungal endophytes were subjected to drought and salinity stress (Moghaddam et al., 2021).

Aiming to find out how these three halotolerant fungal endophytes impacted the establishment of salinity and drought stress tolerance, physiological and biochemical markers, such as biomass, root wet weight, shoot length, leaf chlorophyll concentration, proline content, and antioxidant enzymes activity were assessed. Under salinity and drought stress, barley plants inoculated with three studied fungal endophytes showed greater biomass, root wet weight and shoot length than endophyte-free barley plants. It may be consistent with endophyte-mediated habitat adaptive tolerance (Redman et al., 2011; Moghaddam et al., 2021). One of the main mechanisms of fungal endophytes for decreasing the adverse effects of salinity and drought stress on their host plants is increasing water absorption by increasing root growth (Lata et al., 2018).

Plants’ ability to cope with abiotic stress can be assessed by evaluating leaf chlorophyll levels (Naumann et al., 2008). Salinity and drought stress by inhibiting chlorophyll biosynthesis cause nutritional starvation and suppressed enzyme activities. Under salinity and drought stress, a strong relationship between inoculation of fungal endophytes and enhancement of plant chlorophyll concentration has been indicated (Ghorbani et al., 2018). Here, under salinity and drought stress, barley plants associated with *P. macrospinosa*, *N. goegapense*, *and N. chichastianum* indicated more chlorophyll concentration than endophyte-free controls (Figures 1, 2), suggesting that fungal endophytes have increased barley plants’ tolerance to salinity and drought stress. Similar results were observed in tomato and cucumber plants inoculated with these fungal species (Moghaddam et al., 2021).

Under non-stressed conditions, ROS (Reactive Oxygen Species) are constantly produced in plants but maintained at a non-toxic level. However, ROS accumulation in plants exposed to abiotic stress, such as salinity, drought, high UV radiation, and extreme temperatures can cause significant damage to cell membranes, DNA molecules, and proteins (Elstner, 1991; Tsugane et al., 1999; Apel and Hirt, 2004). To prevent ROS accumulation and suppress their destructive effects, plants produce various anti-oxidative enzymes, such as superoxide dismutase, catalase, peroxidase, and ascorbate peroxidase (Foyer and Noctor, 2000; Bharti et al., 2016). Under environmental stress, endophytic fungi can increase the plant’s antioxidant enzymatic activity to mitigate oxidative damage (Rodriguez and Redman, 2008; Jogawat et al., 2013; Sadeghi et al., 2020; Mahgoub et al., 2021). These compounds, combined with osmotic adjustment, stabilize cell components and eliminate free radicals. The current results show that halotolerant endophytic fungal species *P. macrospinosa* improved the antioxidant enzyme activity in barley under all levels of salinity and drought stress (Figures 4–6). These observations are in accordance with our previous study, indicating that *P. macrospinosa* is capable of increasing the activity of SOD, CAT, and POX enzymes in cucumber and tomato plants (Moghaddam et al., 2021). Barley plants inoculated with *N. goegapense* and, *N. chichastianum* indicated higher levels of antioxidant enzyme activity in comparison to endophyte-free plants under all levels of salinity and drought stress (Figures 4–6). However, these two fungal endophytes increased the activity of SOD, CAT, and POX enzymes of cucumber and tomato plants only in high levels of salinity and drought stress (Moghaddam et al., 2021). This finding suggests that although fungal endophytes improve salinity and drought stress tolerance by increasing the POD, CAT, and POX enzymes activity in their host plant, the potential benefits of these microorganisms are dependent on the host plant’s identity.

Different levels of abiotic stress increase the accumulation of proline in plant tissues (Fahramand et al., 2014; Hashem et al., 2015). Although the molecular role of proline in plant osmotolerance has not yet been demonstrated, it is believed that proline accumulation plays a significant role in plant abiotic stress tolerance via different mechanisms, including detoxification of ROS, osmotic adjustment, and conservation of membrane integrity (Heuer, 2010). Many studies have indicated that endophytic microorganisms can mitigate the destructive effects of salinity and drought stresses by increasing their host plant’s proline content (Waller et al., 2005; Prasad et al., 2013; Mahgoub et al., 2021). In the current investigation, we observed a striking increase of leaf proline content in barley plant inoculated with *P. macrospinosa* under all levels of salinity and drought stress (Figure 3). An increase in proline content infers ionic influx reduction inside the cells and preserves plants by sustaining their osmotic balance. While our previous reports show that *Neocamarosporium* species increase the proline content of cucumber and tomato plants only in higher levels of salinity and drought stress (Moghaddam et al., 2021), here we observe that two halotolerant endophytic fungal species, *N. goegapense*, and *N. chichastianum*, improved the proline content of leaf under all levels of salinity and drought stress.

Overall, the current study and our previous study (Moghaddam et al., 2021) indicated that these three halotolerant endophytic fungi increase both monocot and dicot plant’s resistance to salinity and drought stress across all levels. While *Periconia macrospinosa* increased the activity of antioxidant enzymes and proline content in both monocot and dicot plants under all levels of salinity and drought stress, two *Neocamarosporiun* species did not increase the levels of these biochemical markers in dicot plants under lower levels of the drought and salinity stress. It appears that identical fungal endophytes function differently in different plant species.

## Conclusion

In the present study, three halotolerant endophytic fungal species, *P. macrospinosa, N. goegapense*, and *N. chichastianum*, increased several physiological and biochemical markers, including plant growth parameters, chlorophyll concentrations, antioxidant enzymes activity, and proline content in barley plants exposed to salinity and drought stress. Overall, we observed that these fungi significantly improved plant performance under these abiotic stresses. Such endophytic fungi associated with desert plants have developed some strategies to thrive in the harsh environment of the desert and play a fundamental role in conferring resistance to their host plants against extreme environmental stress. The results of our study show that fungal endophytes might have the ability to increase the abiotic stress tolerance in plants other than their original hosts, which can be utilized in agricultural ecosystems. Different reactions were observed in two *Neocamarosporium* species in inducing tolerance to abiotic stress in dicotyledonous and monocotyledonous plants, highlighting the role of plant identity in such plant-microbe associations. We suggest studying the effects of endophytic fungal species on other major crops, especially in natural agricultural fields. Further in-depth studies are required to understand the molecular changes in the host plants induced by fungi under stressful conditions. Such studies are important regarding the future development of biofertilizers to reduce the excessive utilization of chemical compounds in agricultural fields.

## Data Availability Statement

The original contributions presented in the study are included in the article/Supplementary Material, further inquiries can be directed to the corresponding author/s.

## Author Contributions

MH performed the experiments, analyzed the data, and wrote the manuscript. NS designed and supervised the study. NH-D and SR helped with data analysis and improving the manuscript. This research manuscript was accomplished with the collaboration of all authors.

## Conflict of Interest

The authors declare that the research was conducted in the absence of any commercial or financial relationships that could be construed as a potential conflict of interest.

## Publisher’s Note

All claims expressed in this article are solely those of the authors and do not necessarily represent those of their affiliated organizations, or those of the publisher, the editors and the reviewers. Any product that may be evaluated in this article, or claim that may be made by its manufacturer, is not guaranteed or endorsed by the publisher.

## Supplementary Material

Supplementary Figure 1 Morphological characters of three fungal endophytes reisolated from barley plants. Seven-day colonies of *Neocamarosporium goegapense* **(A)**, *N. chichastianum* **(B)**, *Periconia macrospinosa* **(C)** on Potato Detrose Agar (PDA); Conidiomata and conidia of *N. goegapense* **(D–F)**, and *N. chichastianum* **(G,H)**; Conidiophore and conidia of *P. macrospinosa* **(I–K)**.
