# Supplementary material for: Inducing Tolerance to Abiotic Stress in Hordeum vulgare L. by Halotolerant Endophytic Fungi Associated With Salt Lake Plants
Source: Front Microbiol. 2022 May 20;13:906365. doi: 10.3389/fmicb.2022.906365 (PMC9205400; doi:10.3389/fmicb.2022.906365)
Supplement: Supplementary Figure 1 — Morphological characters of three fungal endophytes reisolated from barley plants. Seven-day colonies of Neocamarosporium goegapense (A), N. chichastianum (B), Periconia macrospinosa (C) on Potato Detrose Agar (PDA); Conidiomata and conidia of N. goegapense (D–F), and N. chichastianum (G,H); Conidiophore and conidia of P. macrospinosa (I–K). [file Image_1.pdf]

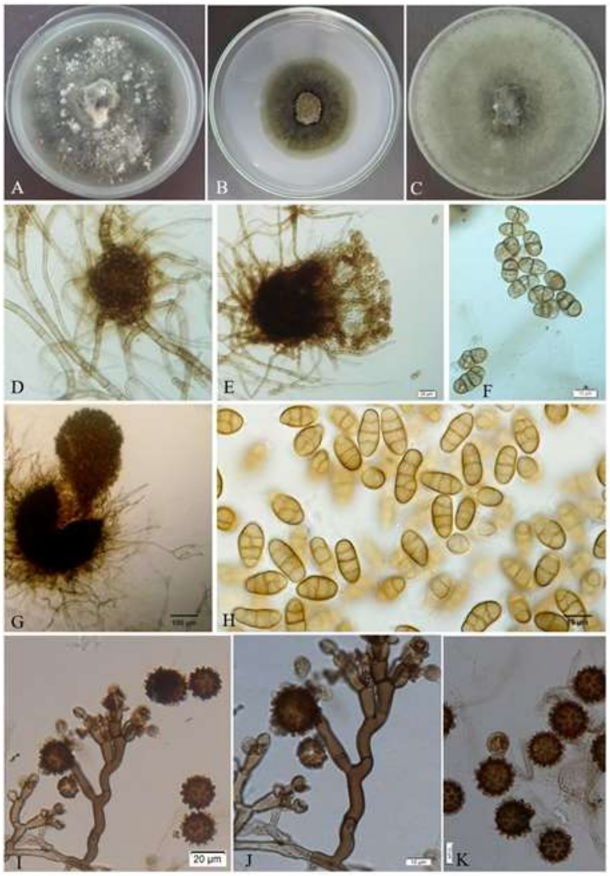

Fig S1. Morphological characters of three fungal endophytes reisolated from barley plants. Seven-day colonies of *Neocamarosporium goegapense* (A), *N. chichastianum* (B), *Periconia macrospinosa* (C) on Potato Detrose Agar (PDA); Conidiomata and conidia of *N. goegapense* (D-F), and *N. chichastianum* (G, H); Conidiophore and conidia of *P. macrospinosa* (I-k).
